# Heparin Thromboprophylaxis in Simultaneous Pancreas-Kidney Transplantation: A Systematic Review and Meta-Analysis of Observational Studies

**DOI:** 10.3389/ti.2023.10442

**Published:** 2023-02-01

**Authors:** Erica Ai Li, Kaveh Farrokhi, Max Y. Zhang, Juliano Offerni, Patrick P. Luke, Alp Sener

**Affiliations:** ^1^ Schulich School of Medicine and Dentistry, Western University, London, ON, Canada; ^2^ Department of Microbiology and Immunology, Schulich School of Medicine and Dentistry, Western University, London, ON, Canada; ^3^ Matthew Mailing Center for Translational Transplant Studies, London, ON, Canada; ^4^ Multi-Organ Transplant Program, London Health Sciences Center, London, ON, Canada; ^5^ Department of Surgery, Division of Urology, London Health Sciences Center, London, ON, Canada

**Keywords:** pancreas transplantation, meta-analysis, heparin, simultaneous pancreas-kidney transplantation, systematic review, thrombosis, thromboprophylaxis

## Abstract

Thrombosis is a leading causes of pancreas graft loss after simultaneous pancreas kidney (SPK), pancreas after kidney (PAK), and pancreas transplant alone (PTA). There remains no standardized thromboprophylaxis protocol. The aim of this systematic review and meta-analysis is to evaluate the impact of heparin thromboprophylaxis on the incidence of pancreas thrombosis, pancreas graft loss, bleeding, and secondary outcomes in SPK, PAK, and PTA. Following PRISMA guidelines, we systematically searched BIOSIS®, PubMed®, Cochrane Library®, EMBASE®, MEDLINE®, and Web of Science® on April 21, 2021. Primary peer-reviewed studies that met inclusion criteria were included. Two methods of quantitative synthesis were performed to account for comparative and non-comparative studies. We included 11 studies, comprising of 1,122 patients in the heparin group and 236 patients in the no-heparin group. When compared to the no-heparin control, prophylactic heparinization significantly decreased the risk of early pancreas thrombosis and pancreas loss for SPK, PAK and PTA without increasing the incidence of bleeding or acute return to the operating room. Heparin thromboprophylaxis yields an approximate two-fold reduction in both pancreas thrombosis and pancreas loss for SPK, PAK and PTA. We report the dosage, frequency, and duration of heparin administration to consolidate the available evidence.

## Graphical Abstract

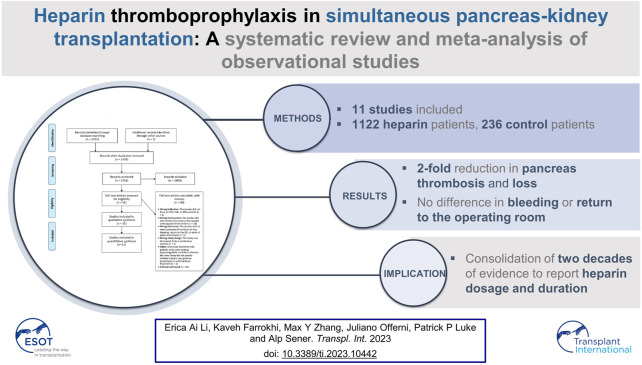

## Introduction

Pancreas transplantation remains the only curative procedure for type 1 diabetes mellitus (T1DM) patients, resulting in long-term control of HbA1c without the risk of serious hypoglycemic events. (1) The first pancreas transplant was performed in 1966 at the University of Minnesota, and since then advancements in immunosuppression, surgical techniques, and surgeon experience have resulted in good overall outcomes for patients (2).

Pancreas transplantation most often occurs simultaneously with kidney transplantation in uremic patients. Simultaneous pancreas-kidney (SPK) transplantation comprises the vast majority of all pancreas transplants, with pancreas after kidney (PAK) being the second most common and pancreas transplant alone (PTA) being the least common. (3, 4) Although T1DM remains the most important indication for pancreas transplantation, there have been a growing number of transplants done in T2DM patients. (5) Other, less frequent indications include transplantation for chronic pancreatitis and after pancreatomy due to malignancy (6).

A common but important complication in pancreas transplantation is thrombosis, which has been reported to have an incidence from 4%–20% (7–9). Thrombosis has been reported as one of the leading causes of pancreas graft loss and technical failure. (10) Despite this, the role of prophylactic anticoagulation remains controversial, with some groups reporting favorable outcomes following anticoagulation and other groups reporting no benefit. (11, 12) There remains no standardized thromboprophylaxis protocol in pancreas transplantation, and transplant centers have different internal protocols and practices. The purpose of this systematic review and meta-analysis is to evaluate the available literature to explore the impact of thromboprophylaxis on the incidence of thrombosis, graft loss, bleeding, and secondary outcomes in SPK, PAK, and PTA.

## Methods

### Data Sources and Search Strategy

The Preferred Reporting Items for Systematic Reviews and Meta-Analysis (PRISMA) guideline was followed to construct this review. We searched the following six databases: BIOSIS^®^, PubMed^®^, Cochrane Library^®^, EMBASE^®^, MEDLINE^®^, and Web of Science^®^. The search strategy is provided in Supplementary Appendix SA1 (Supplemental Digital Content). The search end date was April 21st, 2021. This systematic review and meta-analysis was registered on PROSPERO (CRD42021260585) and may be accessed at: https://www.crd.york.ac.uk/prospero/display_record.php?RecordID=260585.

### Inclusion and Exclusion Criteria

The objective of this review was to assess the outcomes related to prophylactic heparin in SPK, PAK and PTA. We therefore included prospective and retrospective studies written in English and published in peer-reviewed journals in this study. Studies that explored heparin thromboprophylaxis in the intraoperative and/or post-operative period after SPK, PAK or PTA where included. Studies that reported outcomes including incidence of pancreas thrombosis in the early post-transplant period, pancreas graft loss, bleeding episodes, acute return to the operating room (OR), and units of packed red blood cells (pRBC) transfused were included. Given that the inclusion criteria involved heparin as the intervention and thrombosis as the primary outcome, all the included papers reported thromboses that were relevant to the intervention. Early pancreas thrombosis was defined as mention of “early” thrombosis or thrombosis that occurred within 30 days post-transplant. Reviews, editorials, case-reports, conference proceedings, and animal studies were excluded. Studies involving other types of solid organ transplants, where they focused on an intervention other than prophylactic anticoagulation, or where anticoagulation was used to treat diagnosed thrombotic events, were also excluded.

### Study Selection

Studies underwent screening of study titles and abstracts by two reviewers (E.A.L and K.F.) using Rayyan (Rayyan Systems, Boston, United States). The included studies then underwent full-text screening by the same two reviewers. Screening conflicts were reviewed and resolved during meetings with all the reviewers.

### Data Extraction and Quality Assessment

The following data were extracted from the selected articles: title, author(s), journal, study type, transplant type(s), number of patients, mean age, mean BMI, and sex proportion. Recipient specific factors included: mean time of diabetes diagnosis, and mean time on dialysis. Operative factors included: type of anticoagulation, timing of administration (intra/postoperative), dose, and frequency. Donor specific factors included: warm and cold ischemia time. Outcomes included: pancreas thrombosis in the early post-transplant period, pancreas graft loss due to thrombosis, postoperative bleeding incidence, acute return to the operating room, and units of pRBC used. Pancreas thrombosis was defined as any instance where “pancreas thrombosis” was mentioned. Pancreas loss was defined as mention of “pancreas loss” or “pancreatectomy.” Bleeding was defined as mention of “bleed,” “hemorrhage,” or “hematoma”. Acute return to the OR was defined as mention of “re-exploration,” “relaparotomy,” or “thrombectomy.” Data were extracted by two reviewers (E.A.L and K.F) and verified for accuracy and completeness by a different reviewer (M.Y.Z). The Methodological Index for Non-Randomized Studies (MINORS) was used to assess risk of bias of manuscripts (Table 1). (13) The MINORS score examines 12 methodological parameters for non-randomized studies and an additional four parameters for comparative studies. Studies that scored 60% or higher were considered high quality and included.

**TABLE 1:** Characteristics of included studies.

Author, year	Study design	Transplant type	Intervention	Intervention regimen	Intervention group (N = )	Control group (N = )	Intervention group: Mean age (SD)	Control group: Mean age (SD)	Immuno-suppression	MINORS score (%)	Notes
Aboalsamh, 2016	Retrospective	SPK	IV Heparin	IV postoperative heparin 500 IU/h decreased by 100 IH/day over 5 days, then ASA 81 mg/day	29	33	44.0 (1.8)	41.5 (1.5)	ATG, tacrolimus, MMF, methylprednisolone, prednisone	75.0	
Arjona-Sanchez, 2017	Retrospective	SPK	IV Heparin	IV intraoperative sodium heparin, followed by postoperative LMW heparin 40 mg/day for 3 days, then ASA 100 mg/day	127	51	39.1 (7.5)	37.9 (6.4)	NR	75.0	
Fertmann, 2006	Retrospective	SPK	IV Heparin	IV postoperative heparin 2–4 IU/kg	29	—	42.9 (1.6)	—	ATG/ALG, tacrolimus, MMF, methylprednisolone, rapamycin	75.0	Only the cohort treated with heparin alone was used for analysis
Fertmann, 2011	Retrospective	PAK + PTA	IV Heparin	IV postoperative heparin 2–4 IU/kg according to Hb, partial thromboplastin time	13	—	35.7 (2.2)	—	ATG/ALG, tacrolimus, MMF, cyclosporine, methylprednisolone, "steroids"	75.0	Only the cohort treated with heparin alone was used for analysis
Humar, 2001	Retrospective	SPK	IV Heparin	IV postoperative heparin 300–500 U/h for 5 days, then ASA for 3 months	193	—	37.3 (NR)	—	ALG/daclizumab, tacrolimus, MMF, prednisone	75.0	
		PAK	IV Heparin	IV postoperative heparin 300–500 U/h for 5 days, then ASA for 3 months	205	—	40.2 (NR)	—	ALG/daclizumab, tacrolimus, MMF, prednisone		
Kim, 2012	Retrospective	SPK	SC Heparin	IV intraoperative heparin 70 U/kg, followed by SC heparin 3,000–5000 U with intention to aPTT prolongation, followed by ASA during admission	67	—	—	—	—	75.0	
		PAK	SC Heparin		10	—	—	—	—		
		PTA	SC Heparin		42	—	—	—	—		
Raveh, 2019	Retrospective	SPK + PAK + PTA	IV Heparin	IV intraoperative heparin infusion targeting an aPTT of 45–50s	32	10	—	—	ATG, basiliximab, methylprednisolone	79.2	
Scheffert, 2014	Retrospective	SPK + PAK + PTA	IV Heparin	IV postoperative heparin 200–400 U/h for 33–68h, followed by ASA 300 mg *per rectum*/day, then oral ASA 325 mg/day	52	100	41 (7.5)	40.0 (8.0)	ALG/IL-2 agonist, tacrolimus, MMF, cyclosporine, prednisone	79.2	90% of patients were given heparin within the first 24 h postoperative period. The median (IQR) heparin dose was 300 (200–400) IU/h, or 5 (3.4–6) IU/kg/h, without titrating to a goal activated partial thromboplastin time. The median (IQR) duration was 48 (33–69) hours
Schenker, 2009	Retrospective	SPK + PAK + PTA	SC or IV Heparin	Either postoperative SC LMW heparin 3,000–3800 IU/day or postoperative IV heparin 400–600 U/h for 9 ± 4.9 days, followed by SC LMW heparin	188	—	41.6 (8.2)	—	ATG, tacrolimus, MMF, prednisolone, sirolimus	75.0	
Shin, 2014	Retrospective	SPK + PAK + PTA	SC or IV Heparin	Either SC postoperative heparin 3,000–5000 U every 8 h or IV postoperative heparin 400–1000 U/h, followed by oral warfarin for 3 months	135	—	37.0 (9.0)	—	ATG, basiliximab, tacrolimus, MMF, prednisolone, "steroids	75.0	
Stratta, 2014	Retrospective	PTA	IV heparin	IV intraoperative heparin 2000–3000 U (30–50 U/kg), followed by IV postoperative heparin 300U/h for 1 day, then 400U/h for 1 day, then 500U/h until post-op day 5	40	—	42.2 (8.7)	—	ATG, tacrolimus, MMF, "corticosteroids"	75.0	The SPK group was excluded from analysis because only 37 out of 162 SPK patients received heparin

ALG, anti-lymphocyte globulin; ASA, acetylsalicylic acid; ATG, anti-thymocyte globulin; IV, intravenous; MMF, mycophenolate mofetil; NR, not reported; SC, subcutaneous.

### Statistical Analysis

Two methods of data synthesis were performed to account for differences in study methodology. First, we conducted meta-analysis of the comparative studies involving a no-heparin control using Review Manager (RevMan, Version 5.4, The Nordic142 Cochrane Center, Copenhagen, Denmark). Study heterogeneity was examined using the I^2^ statistic. An I^2^ < 50% suggested low study heterogeneity and a fixed-effect model was used, whereas an I^2^ > 50% suggested high study heterogeneity and a random-effects model was used. Results were visualized as forest plots. Publication bias was assessed using funnel plots for each outcome.

Second, we pooled the populations from the comparative and non-comparative studies to allow for comparison of demographic, intraoperative, and postoperative characteristics between the heparin and no-heparin groups. Statistical analysis was performed using R (Version 4.1.2., Boston, United States) and GraphPad QuickCalcs (GraphPad Software, Inc., California, United States). Two-tailed Fisher’s exact tests were conducted for the categorical variables and results were reported as incidence (percentage of total). Unpaired t-tests were conducted for the continuous variables and results were reported as mean (SD). Cohen’s kappa coefficient was obtained to assess inter-rater reliability. A *p*-value ≤0.05 was considered statistically significant.

## Results

### Search Results

**FIGURE 1:**
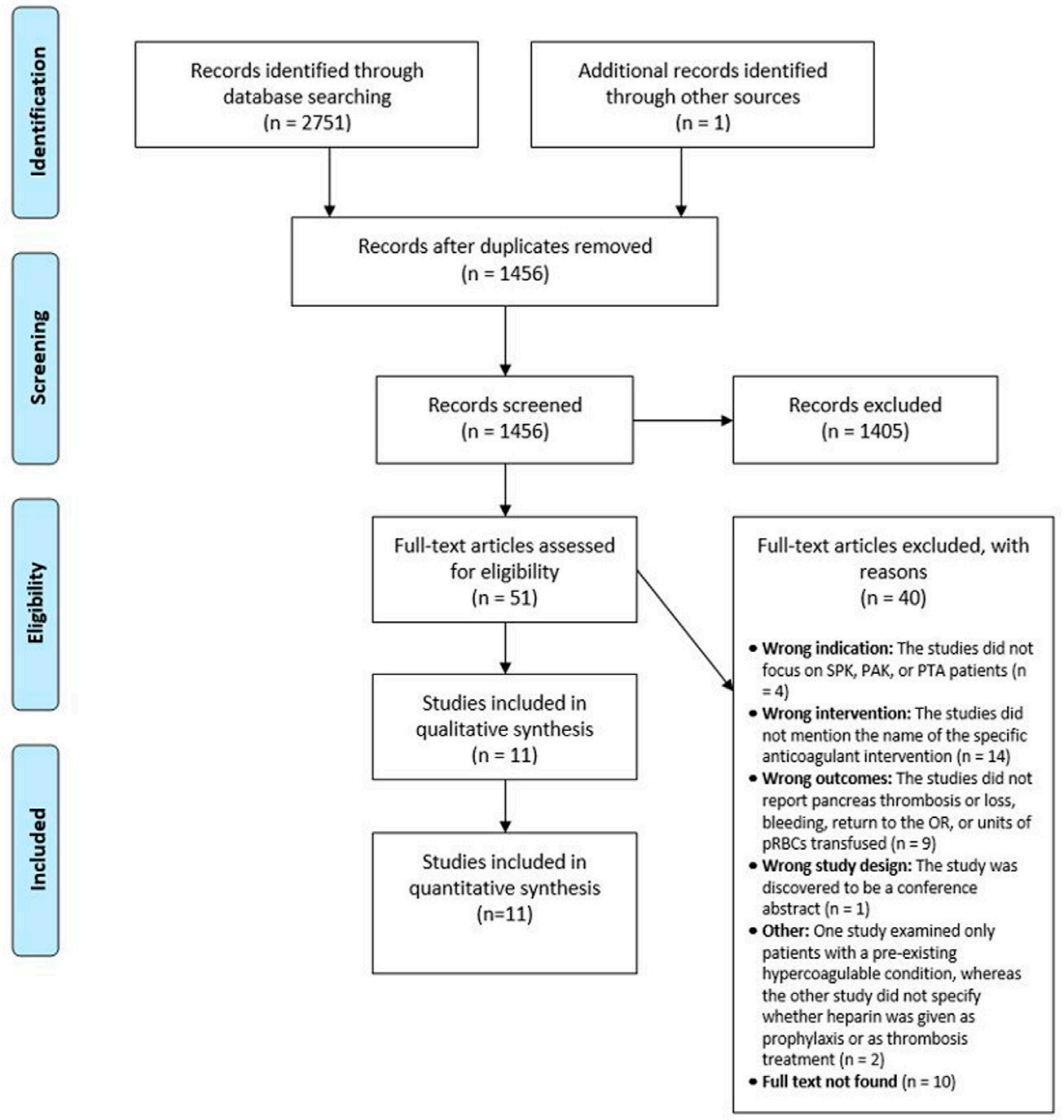
PRISMA Flow diagram.

The study inclusion process is summarized in the PRISMA flow diagram (Figure 1). After removal of duplicates, 1,456 studies underwent Level 1 abstract and title screening, of which 51 studies were eligible for Level 2 full-text screening. Eleven studies were ultimately included for quantitative synthesis (11, 12–22). All 11 studies were retrospective. Level 1 screening had moderate agreement with a Cohen’s kappa of 0.56 and Level 2 screening had substantial agreement with a Cohen’s kappa of 0.67.

### Study Characteristics

Characteristics of the included studies are summarized in Table 1. These included studies comprised of a total of 1,358 patients, with 1,122 patients in the intervention group and 236 patients in the control group. Mean recipient age was 40.4 (7.85) for the heparin group and 39.7 (6.90) for the control group, with no significant difference detected (*p* = 0.96). There were three studies involving only SPK transplant patients, (11, 12, 17) one study with both PAK and PTA patients; (16) one study with both SPK and PAK patients; (22) and five studies combining SPK, PAK, and PAK patients; (14, 18–21). There was also one study involving both SPK and PTA patients. However, only the PTA data was suitable for quantitative synthesis (Table 1) (15).

### Study Quality and Publication Bias

Methodological quality of the included studies is summarized in Table 1 and in Supplementary Appendix SA2 (see Supplemental Digital Content). All studies lacked prospective data collection and prospective calculation of the study size. However, all studies possessed a clearly stated aim, with appropriate study endpoints. There were five comparative studies involving a no-heparin control, and these studies had baseline equivalence between groups and adequate statistical analysis.

Funnel plots were assessed for publication bias, with one funnel plot for each outcome of interest. We did not observe any overt asymmetry or pattern in the funnel plots for the incidence of thrombosis, graft loss, bleeding, acute return to the OR, or mean units of pRBCs transfused (see Supplemental Digital Content, Supplementary Appendix SA3).

### Analysis of Comparative Studies

#### Heparin Reduced the Incidence of Pancreas Thrombosis

**FIGURE 2:**
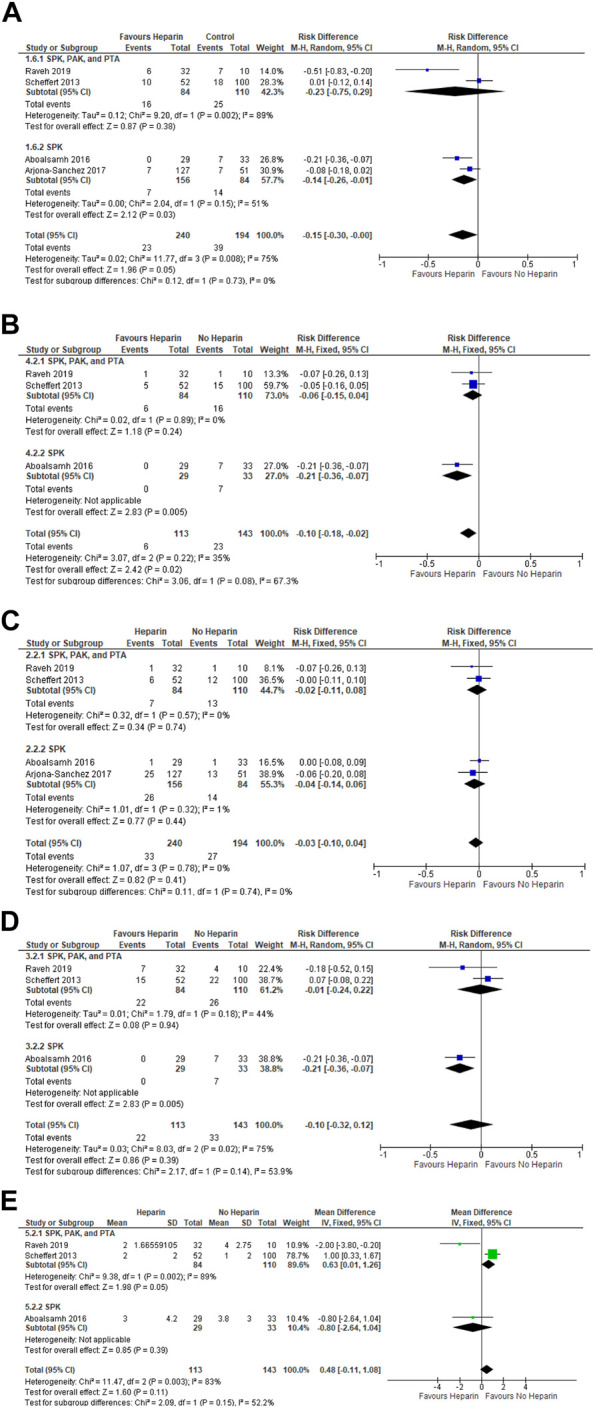
Forest plots examining the impact of heparin on the **(A)** incidence of pancreas thrombosis, **(B)** incidence of pancreas loss due to thrombosis, **(C)** incidence of postoperative bleeding, **(D)** incidence of acute return to the OR, **(E)** mean units of pRBCs transfused.

There were four studies available for analysis, comprising of 240 patients in the heparin treatment group and 194 patients in the control group. (11, 12, 18, 20) Overall, there was a significantly lower incidence of pancreas graft thrombosis in the heparin group compared to the no-heparin group, with a risk difference of −0.15 (95% CI = −0.30, −0.00; *p* = 0.05) (Figure 2A). Subgroup analysis revealed no significant difference in pancreas thrombosis between treatment and control groups when looking at the studies that mixed their SPK, PAK, and PTA patients together (Risk difference = −0.23; 95% CI = −0.75, 0.29; *p* = 0.38). However, there was a significantly lower incidence of early pancreas thrombosis in the heparin group compared to the control group when looking at the studies that only included SPK patients (Risk difference = −0.14; 95% CI = −0.26, −0.01; *p* = 0.03). In fact, the total incidence of pancreas thrombosis in the heparin group was less than half of that in the no-heparin group.

#### Heparin Reduced the Incidence of Pancreas Loss

Three studies were available for analysis, consisting of 113 patients in the heparin group and 143 patients in the no-heparin control group. (12, 18, 20) When assessing the overall effect, there was a significantly lower incidence of pancreas loss due to graft thrombosis in the heparin group compared to the control group (Risk difference = −0.10; 95% CI = −0.18, −0.02; *p* = 0.02) (Figure 2B). When examining the subgroup consisting of SPK, PAK, and PTA patients, there was no significant difference between groups in incidence of pancreas loss (Risk difference = −0.06; 95% CI = −0.15, 0.04; *p* = 0.24). For the SPK subgroup, there was a significantly lower incidence of pancreas loss in the heparin group (Risk difference = −0.21; 95% CI = −0.36, −0.07; *p* = 0.005).

#### Heparin did Not Impact Postoperative Incidence of Bleeds, Incidence of Acute Return to the OR, or Units of pRBC Used

Four studies were available for analysis of the incidence of bleeding, consisting of 240 patients in the heparin group and 194 patients in the no-heparin control group. (11, 12, 18, 20) Overall, there was no significant difference in the incidence of bleeding between groups (Risk difference = −0.03; 95% CI = −0.10, 0.04; *p* = 0.41) (Figure 2C). Subgroup analysis also revealed no differences in the incidence of bleeds in neither the combined transplant subgroup (Risk difference = −0.02; 95% CI = −0.11, 0.08; *p* = 0.74), nor the SPK subgroup (Risk difference = −0.04; 95% CI = −0.14, 0.06; *p* = 0.44).

Three studies were available for analysis of the incidence of acute return to the OR and the mean number of packed RBCs used. (12, 18, 20) There were 113 patients in the heparin group and 143 patients in the control group (Figures 2D,E). Overall, there was no significant difference in the incidence of acute return to the OR between the heparin and no-heparin groups (Risk difference = −0.10; 95% CI = −0.32, 0.12; *p* = 0.39). There were no significant differences when examining the combined SPK, PAK, PTA subgroup (Risk difference = −0.01; 95% CI = −0.24, 0.22; *p* = 0.90). There was a significantly lower risk of acute return to the OR in the SPK subgroup (Risk difference = −0.21; 95% CI = −0.36, −0.07; *p* = 0.005).

Furthermore, there was overall no significant difference in units of pRBCs used between the heparin and no-heparin groups (Mean difference = 0.48; 95% CI = -0.11,1.08; *p* = 0.11). Subgroup analysis also revealed no significant differences in the combined SPK, PAK, PTA subgroup (Mean difference = 0.63; 95% CI = 0.01, 1.26; *p* = 0.05) and the SPK subgroup (Mean difference = −0.80; 95% CI = −2.64, 1.04; *p* = 0.39).

### Analysis of Both Comparative and Non-Comparative Studies

#### SPK Only

There were studies that met inclusion criteria that lacked a no-heparin control. To incorporate these studies, we grouped their patient cohorts with the heparin cohorts from the comparative studies. The no-heparin group derived from the comparative studies was used as the control. When examining the SPK subgroup, there were five studies available for analysis, (11, 12, 17, 19, 22) with two of these studies being comparative studies. (11, 12) For the study by Fertmann et. al. 2006, only the cohort treated with heparin alone was used for analysis (Table 1). Donor mean age, donor mean BMI, mean cold ischemia time, and mean warm ischemia time were significantly higher in the heparin group compared to the control group (Table 2). The rate of thrombosis and pancreas loss due to thrombosis were significantly lower in the heparin group compared to the no-heparin control group. There was no significant difference in the incidence of bleeding, acute return to the OR, or units of pRBCs transfused between groups.

**TABLE 2:** Analysis of SPK studies.

	Total N	Heparin		Total N	No Heparin		*p*
		Mean (SD) or incidence (%)			Mean (SD) or incidence (%)		
Demographic Characteristics							
Recipient Mean Age	209	40.9	(6.33)	84	39.3	(5.32)	0.045
Recipient Mean BMI	156	23.0	(2.62)	84	23.3	(2.18)	0.32
Recipient Incidence Male	349	231 (66.2%)	(9.00)	84	57 (67.9%)	(7.75)	0.80
Mean Time Diabetes (Years)^a^	127	24.0	(6.00)	51	24.0	(4.8)	1.00
Mean Time Dialysis (Months)^a^	29	33.2		33	33.9		0.61
Donor Mean Age^a^	82	32.6	(2.24)	33	31.1	(2.20)	0.0015
Donor Mean BMI^a^	29	25.9	(0.90)	33	24.6	(0.60)	<0.001
Donor Proportion Male^a^	222	134 (60.4%)		33	16 (48.5%)		0.26
Intraoperative Characteristics							
Mean Cold Ischemia Time (h)	156	10.8	(2.72)	84	9.4	(2.49)	<0.001
Mean Warm Ischemia Time (h)^a^	29	0.5	(0.03)	33	0.4	(0.00)	<0.001
Postoperative Complications							
Thrombosis	469	43 (9.2%)		83	14 (16.9%)		0.048
Pancreas loss due to thrombosis^a^	96	1 (1.0%)		33	7 (21.2%)		0.0003
Bleeding	349	92 (26.4%)		84	14 (16.7%)		0.07
Return to the OR^a^	222	50 (22.5%)		33	14 (16.9%)		1.00
Units of pRBC^a^	29	3.0	(4.20)	33	3.8	(3.00)	0.39

^a^ Only one study available.

#### Analysis of all Included Studies: SPK, PAK and PTA

Eleven studies were available for analysis (11, 12, 14–22), of which four were comparative studies. (11, 12, 18, 20) For the study by Fertmann et. al. 2011, only the cohort treated with heparin alone was used for analysis (Table 1). Both Fertmann studies were included because two different patient cohorts were used for each study. Fertmann 2006 included only SPK patients, whereas Fertmann 2011 included only PAK and PTA patients. The combined cohort consisted of SPK, PAK, and PTA patients. There were significantly more male patients in the no-heparin group compared to the heparin group (Table 3). The rate of thrombosis and pancreas loss due to thrombosis were significantly lower in the heparin group. Furthermore, there was no significant difference in the incidence of bleeding or return to the OR but there was a significantly higher mean number of units of pRBC transfused in the heparin group compared to the no-heparin group.

**TABLE 3:** Analysis of all included studies.

	Total N	Heparin		Total N	No Heparin		*p*
		Mean (SD) or incidence (%)			Mean (SD) or incidence (%)		
Demographic Characteristics							
Recipient Mean Age	655	40.4	(7.85)	184	39.7	(6.90)	0.96
Recipient Mean BMI	343	22.3	(2.77)	184	23.7	(2.67)	0.74
Recipient Incidence Male	860	531 (61.7%)	(8.6)	184	114 (62.0%)	(7.75)	1.00
Mean Time Diabetes (Years)	489	25.4	(24.75)	51	24.0	(4.80)	0.96
Mean Time Dialysis (Months)^a^	217	28.2	(7.85)	33	33.9		0.93
Donor Mean Age	497	30.6	(11.00)	133	25.0	(8.62)	0.80
Donor Mean BMI	444	22.8	(3.71)	133	24.9	(8.67)	0.80
Donor Proportion Male	802	453 (56.5%)		133	91 (68.4%)		0.010
Intraoperative Characteristics							
Mean Cold Ischemia Time (h)	436	11.9	(3.48)	184	11.2	(3.53)	0.91
Mean Warm Ischemia Time (h)	81	0.55	(0.18)	133	0.5	(0.14)	0.73
Postoperative Complications							
Thrombosis	1,204	163 (13.5%)		194	39 (20.1%)		0.0205
Pancreas loss due to thrombosis	586	43 (7.3%)		143	23 (16.1%)		0.0029
Bleeding	788	148 (18.8%)		194	27 (13.9%)		0.12
Return to the OR	661	142 (21.5%)		143	33 (23.1%)		0.66
Units of pRBC	144	2.5	(2.53)	143	1.9	(2.65)	0.044

^a^ Only one study available.

#### Analysis of Contemporary Studies: SPK, PAK and PTA

Lastly, we explored the effect of heparin when including only contemporary works published within the past 10 years. The analysis of comparative studies conducted in the previous section involved only contemporary studies from 2013 and onward. When combining both the comparative and non-comparative studies, seven papers were available for analysis, which included studies from 2012 and onward (11, 12, 14–20). Four of these studies were comparative studies. Analysis of these contemporary works revealed similar trends to that of the analysis of all included studies (Table 4).

**TABLE 4:** Analysis of contemporary studies from the past 10 years.

	Total N	Heparin		Total N	No Heparin		*p*
		Mean (SD) or incidence (%)			Mean (SD) or incidence (%)		
Demographic Characteristics							
Recipient Mean Age	502	37.9	(8.92)	(8.92)	39.7	(6.90)	0.91
Recipient Mean BMI	343	22.3	(2.77)	(2.77)	23.7	(2.67)	0.74
Recipient Incidence Male	502	287 (57.2%)	(9.2)	(9.2)	114 (62.0%)	(7.75)	0.29
Mean Time Diabetes (Years)	421	20.5	(6.0)	(6.0)	24.0	(4.80)	0.90
Mean Time Dialysis (Months)^a^	29	33.2	(8.92)	(8.92)	33.9		0.61
Donor Mean Age	375	28.5	(10.52)	133	25.0	(8.62)	0.85
Donor Mean BMI	375	22.1	(3.87)	133	24.9	(8.67)	0.74
Donor Proportion Male	335	184 (54.9%)		133	91 (68.4%)		0.0091
Intraoperative Characteristics							
Mean Cold Ischemia Time (h)	367	10.3	(4.19)	184	11.2	(3.53)	0.89
Mean Warm Ischemia Time (h)	81	0.55	(0.18)	133	0.5	(0.14)	0.83
Postoperative Complications							
Thrombosis	534	85 (15.9%)		194	39 (20.1%)		0.18
Pancreas loss due to thrombosis	367	10 (2.7%)		143	23 (16.1%)		<0.0001
Bleeding	359	54 (15.0%)		194	27 (13.9%)		0.80
Return to the OR	232	47 (20.3%)		143	33 (23.1%)		0.52
Units of pRBC	113	2.3	(2.68)	143	1.9	(2.65)	0.23

^a^ Only one study available.

## Discussion

Graft thrombosis remains a leading cause of pancreas graft loss after SPK, PAK, and PTA. Despite this, the evidence for heparin thromboprophylaxis is mixed and there is great variability in the regimens used. We systematically reviewed the available literature to investigate the effect of prophylactic heparin on pancreas graft thrombosis and loss, as well as on other postoperative complications. We conducted two methods of quantitative synthesis: the first involving analysis of the included comparative studies, and the second involving analysis of both comparative and non-comparative studies. The first method of analysis revealed that heparin significantly reduced the overall incidence of early pancreas thrombosis and pancreas loss, without impacting the incidence of acute return to the OR, bleeding, or units of pRBC transfused. The second method of analysis revealed similar findings for SPK, PAK, and PTA, with an increase in the mean units of pRBCs transfused in the heparin group compared to the no-heparin group.

Analysis of comparative studies revealed greater overall effects and lower study heterogeneity in the SPK subgroup compared to the combined SPK, PAK, and PTA subgroup. This was evident for the pancreas thrombosis outcome. Previous studies report differential rates of pancreas thrombosis and pancreas loss for SPK, PAK and PTA, (16, 23) which when combined as a subgroup, may offset any true trends. Even so, we found that the overall incidence of pancreas thrombosis was over two times lower in the heparin group, while the overall incidence of pancreas loss was over three times lower in the heparin group. Because SPK requires greater time and technical involvement, the impact of heparin may be especially evident in this subgroup. With pancreas thrombosis being a common cause of pancreas loss, (8) heparin has an appreciable effect on improving graft survival, particularly after SPK transplantation.

Analysis of both comparative and non-comparative SPK studies showed a significantly higher mean donor age, mean donor BMI, mean cold ischemia time, and mean warm ischemia time in the heparin group. These variables have been previously shown to be associated with graft thrombosis. (24) Heparin remained effective in reducing the incidence of thrombosis and pancreas loss despite this group possessing factors associated with pancreas thrombosis. For this review, caution is warranted when interpreting these demographic differences because for three variables, there was only one study in the no-heparin group. The observed statistical significance may then be a by-product of the heterogeneity in the reporting of demographic data between the intervention group and control group. Furthermore, some pancreas transplant centers have avoided the use of systemic anticoagulation to prevent bleeding complications. (25, 26) However, a number of transplant groups note that graft thrombosis and loss are more detrimental than bleeds that can be controlled by transfusion or laparotomy. (27, 28) Importantly, our analysis shows that in SPK transplants, the beneficial effects of heparin did not increase the risk of acute bleeding requiring laparotomy or use of blood transfusions.

When examining all the included comparative and non-comparative studies, we confirm that heparin is associated with a significant reduction in thrombosis and pancreas loss for SPK, PAK, and PTA. In this combined analysis, patients treated with heparin were transfused with a mean of 0.62 units of additional pRBC compared to the control—which although is statistically significant—is not clinically significant. We may attribute this statistical difference to the large sample sizes and relatively small standard deviations for both groups. By incorporating all the included studies, we also balance the demographic differences between the heparin group and no-heparin group, without observing any changes in the efficacy of heparin in mitigating early post-transplant thrombosis and pancreas loss.

This analysis is limited by the lack of prospective, randomized controlled studies, as well as by the inclusion of only English studies. The available literature presents with higher degrees of confounding and greater variability in reporting, which may limit the conclusions drawn. A limitation with retrospective data is that we lack insight on the decision-making process behind which patients were heparinized. Even in comparative studies, there exists patient-specific factors—such as a history of a clotting disorder or a prolonged cold ischemia time—which may influence the decision for heparin thromboprophylaxis. (15) Changes in personnel, surgical technique, and postoperative management in institutions over time may also impact patient outcomes. Of the included studies, there is also great variability in the timing and dosage of heparin administered. This may be the result of evolving institutional practices and a lack of consistent evidence on prophylactic heparin usage. Additionally, there are technical factors that may influence the risk of thrombosis, such as the type of exocrine drainage. Well-established evidence indicates that bladder drainage may confer long-term urologic and metabolic complications, with contemporary practice utilizing enteric drainage, or occasionally portal-enteric drainage. (29) We controlled for these institutional and technical factors by analyzing only the included contemporary studies. Given that the impact of heparin on post-operative outcomes remained the same even with this contemporary analysis, the effects of heparin may be robust enough to withstand evolving institutional and surgical practices.

This review highlights the gaps in the literature, while providing a synthesis of the data that is available to us at this current time. To our knowledge, this is the first systematic review in existence that explores the impact of heparin thromboprophylaxis in SPK, PAK, and PTA. The pooled sample sizes for the heparin and no-heparin groups are sufficiently large such that the assumption of normal sampling distribution may be fulfilled by the Central Limit Theorem. (30) The large sample sizes help account for the probability of error from the above limitations. Future research that prospectively compares the impact of heparin to that of a no-heparin control is warranted. Given that all the comparative studies in this review were published within the last 10 years, this suggests an influx of higher quality studies exploring this subject in recent times. We foresee more high-quality studies will become published in the near future, which will warrant additional meta-analyses.

**FIGURE 3:**
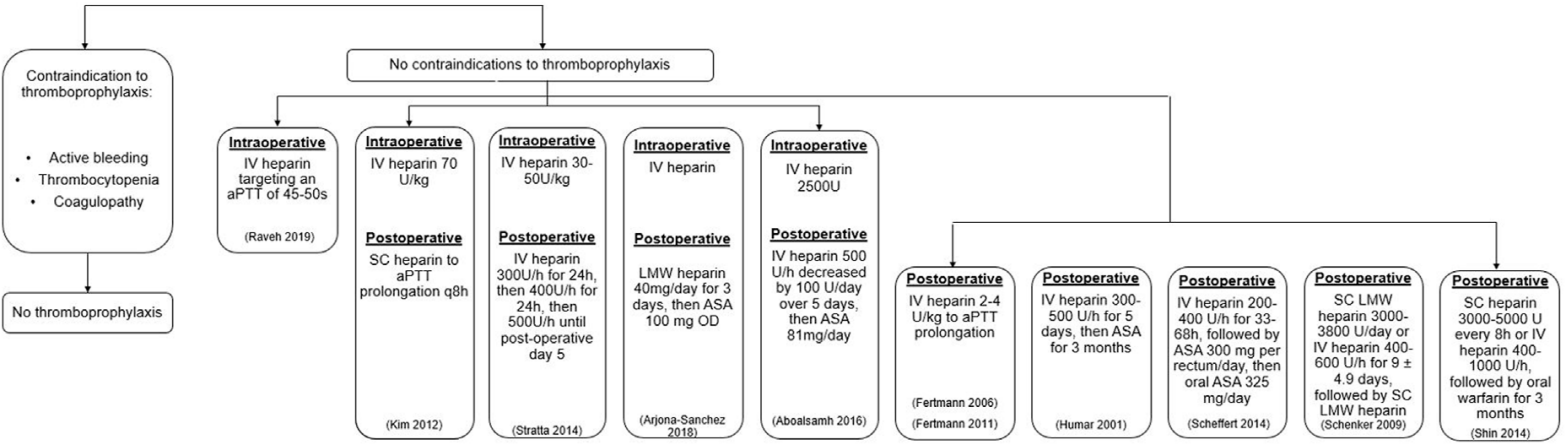
Flow diagram of the prophylactic heparin regimens presented in this review. ASA, acetylsalicylic acid; IU, international unit; IV, intravenous; SC, subcutaneous.

Based on the findings of this study, we present a flow diagram outlining the available treatment regimens (Figure 3). In the absence of contraindications to heparin thromboprophylaxis, intraoperative intravenous heparin 30–70 IU/kg was used. (15, 19) During the postoperative period, either subcutaneous heparin 3,000–5000 IU 1–2 times per day (14, 19, 21) or intravenous heparin infusion 200–1000 IU/h for 1–14 days (12, 14–17, 20–22) were reported. Subsequent maintenance with aspirin 81–325 mg daily was then used (11, 12, 20).

## Conclusion

We demonstrate that prophylactic heparinization produces over a two-fold reduction in early pancreas thrombosis and pancreas loss for SPK, PAK and PTA, without increasing the incidence of bleeding or acute return to the OR. With early postoperative complications, such as pancreas graft thrombosis, persisting as a leading cause for graft loss, heparin thromboprophylaxis holds promise for enhancing graft survival without imposing additional postoperative complications. This meta-analysis culminates 2 decades of available evidence to highlight the efficacy of heparin thromboprophylaxis for improving graft survival for SPK, PAK, and PTA patients.

## Abbreviations

IU, International unit; PAK, Pancreas after kidney transplant; PTA, Pancreas transplant alone; SPK, Simultaneous pancreas-kidney transplant.
