# Supplementary material for: Heparin Thromboprophylaxis in Simultaneous Pancreas-Kidney Transplantation: A Systematic Review and Meta-Analysis of Observational Studies
Source: Transpl Int. 2023 Feb 1;36:10442. doi: 10.3389/ti.2023.10442 (PMC9928749; doi:10.3389/ti.2023.10442)

Appendix 1. Search strategy.

Related words for kidney transplantation	("Kidney Transplant*"OR "renal transplant*" OR "Kidney graft*" OR "Renal graft" OR "kidney allograft*" OR "Renal allograft*")
	AND
Related words for pancreas transplant	(“pancreas*” OR “Pancreas Transplant*” OR “islet transplant*” OR “SKPT” OR “SPKT” OR “Pancreas allograft*” OR “Pancreas graft*” OR “Simultaneous Kidney Pancreas*” OR “Simultaneous Pancreas Kidney*”)
	AND
Related words for Heparin	("Heparin*" OR "Low Molecular Weight Heparin" OR "anticoagulant*" OR coagulation* OR Thrombo* or anticoag* or anti-coag*)

Appendix 2. Methodological Quality of Included Studies by MINORS Criteria.

Study, Year	Type of Study	Score (%)	**Methodological terms for non-randomised studies**								**Additional criteria in the case of comparative studies**			
			1. A clearly stated aim	2. Included consecut-ive patients	3. Prospect-ive collection of data	4. Endpoints approp-riate to study aim	5. Unbiased assess-ment of study endpoint	6. Follow-up period approp-riate to study aim	7. Loss to follow up less than 5%	8. Prospect-ive calculat-ion of study size	9. An adequate control group	10. Contemp-orary groups	11. Baseline equival-ence of groups	12. Adequate statistical analyses
Aboalsamh, 2016	Retrospective	75.00	2	2	0	2	2	2	2	0	2	1	1	2
Arjona-Sanchez, 2017	Retrospective	75.00	2	1	0	2	2	2	2	0	2	1	2	2
Fertmann, 2006	Retrospective	75.00	2	2	0	2	2	2	2	0				
Fertmann, 2011	Retrospective	75.00	2	2	0	2	2	2	2	0				
Humar, 2001	Retrospective	75.00	2	2	0	2	2	2	2	0				
Kim, 2012	Retrospective	75.00	2	2	0	2	2	2	2	0				
Raveh, 2019	Retrospective	79.17	2	2	0	2	2	2	2	0	2	2	1	2
Scheffert, 2014	Retrospective	79.17	2	2	0	2	2	2	2	0	2	1	2	2
Schenker, 2009	Retrospective	75.00	2	2	0	2	2	2	2	0				
Shin, 2014	Retrospective	75.00	2	2	0	2	2	2	2	0				
Stratta, 2014	Retrospective	75.00	2	2	0	2	2	2	2	0				

Appendix 3. Funnel plots of the studies included for meta-analysis. Each funnel plot encompasses the studies for each outcome. A) Incidence of early pancreas thrombosis, B) incidence of pancreas loss due to thrombosis, C) Incidence of postoperative bleeding, D) Incidence of acute return to the OR, E) Mean units of pRBCs transfused.

A)

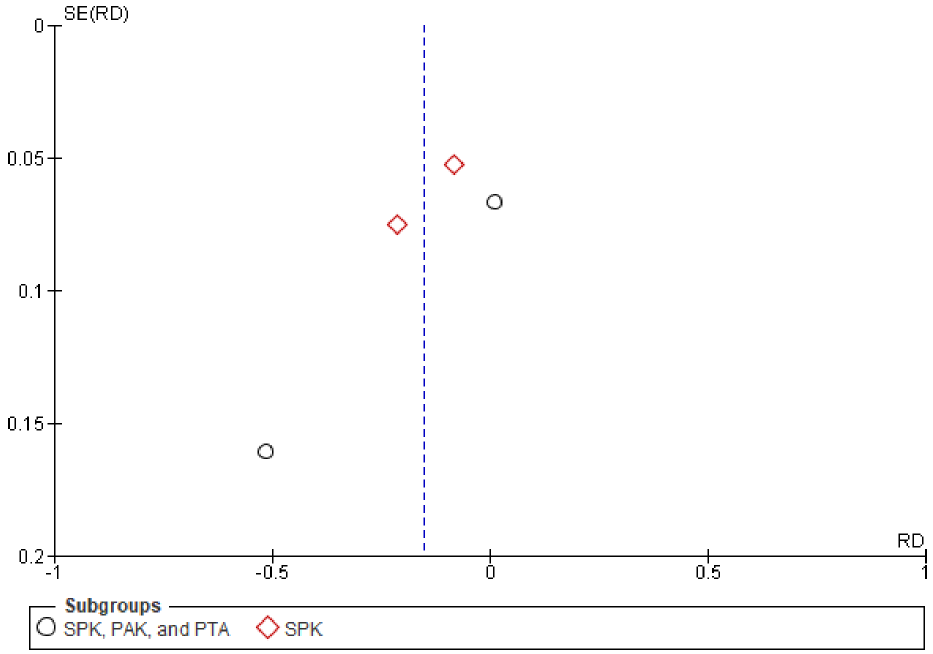

B)

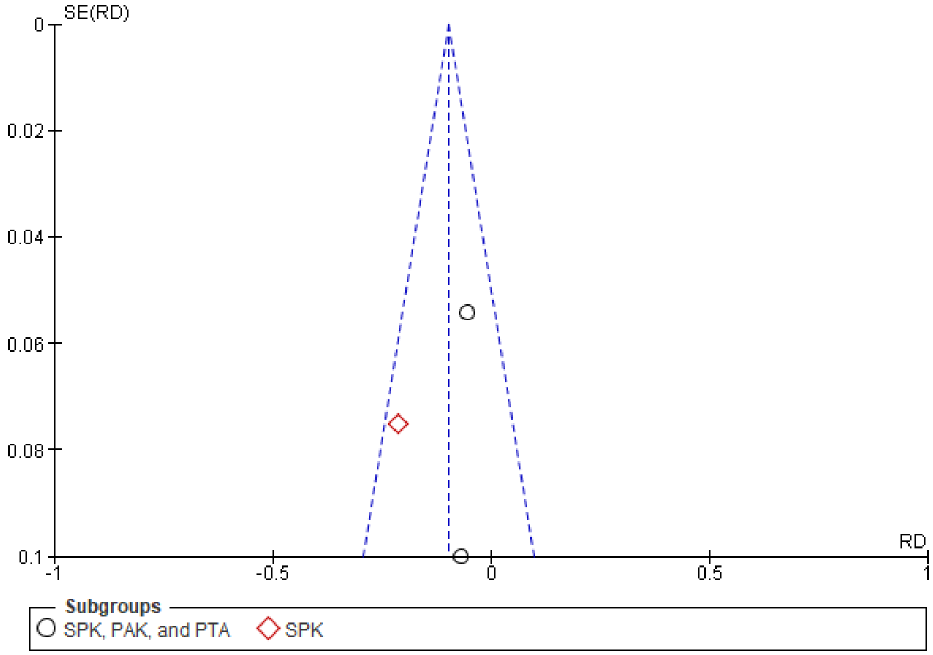

C)

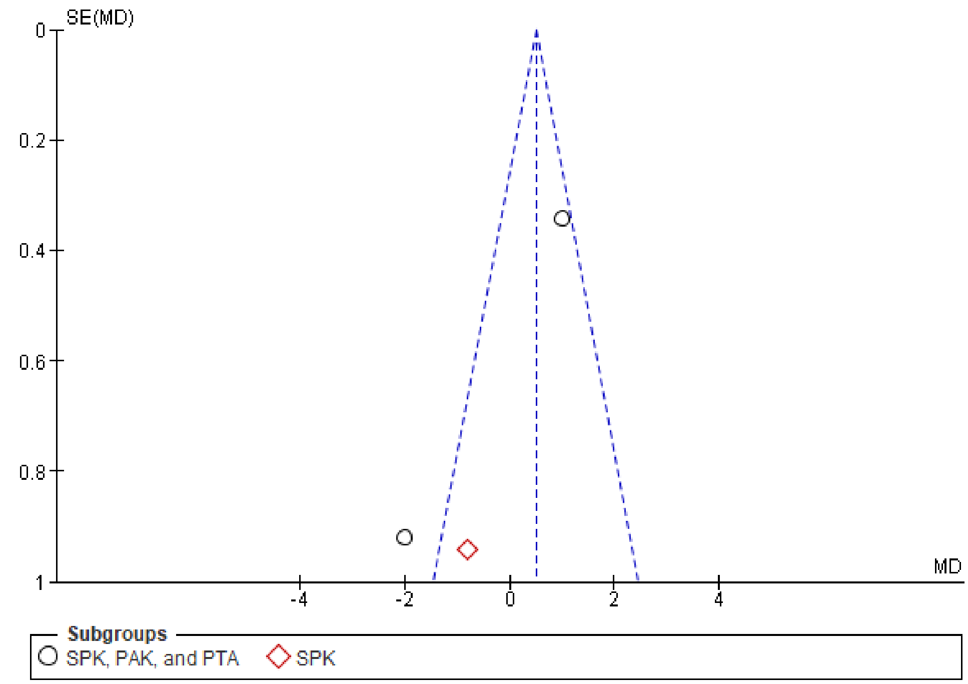
SE(MD)
0
0.2
0.4
0.6
0.8
1
MD
-4
-2
0
2
4
Subgroups
SPK, PAK, and PTA
SPK

D)

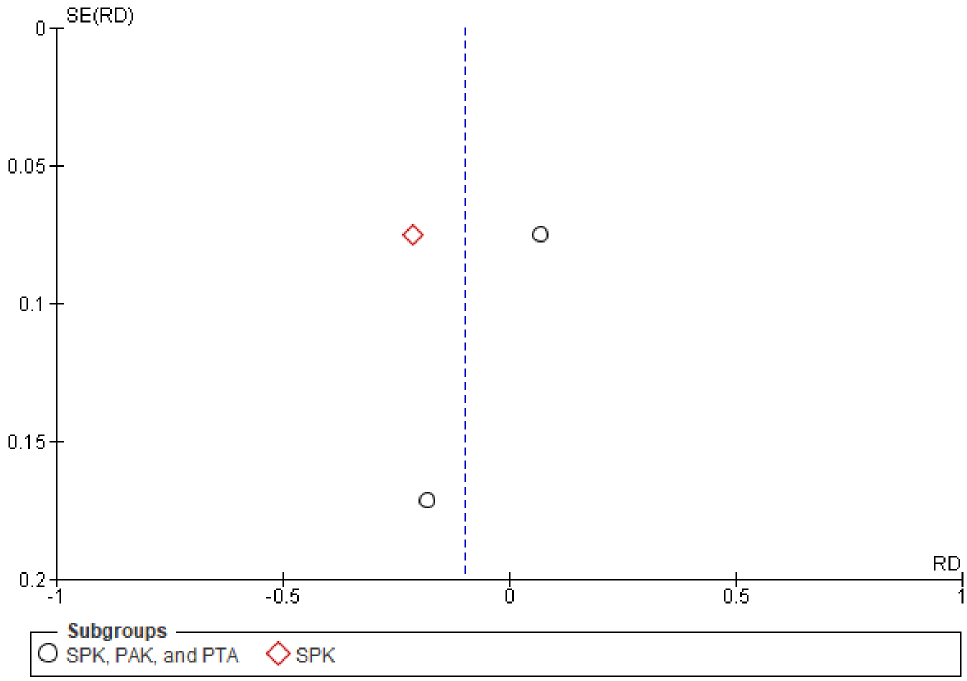
SE(RD)
0
0.05
0.1
0.15
0.2
RD
-1
-0.5
0
0.5
1
Subgroups
SPK, PAK, and PTA
SPK

E)

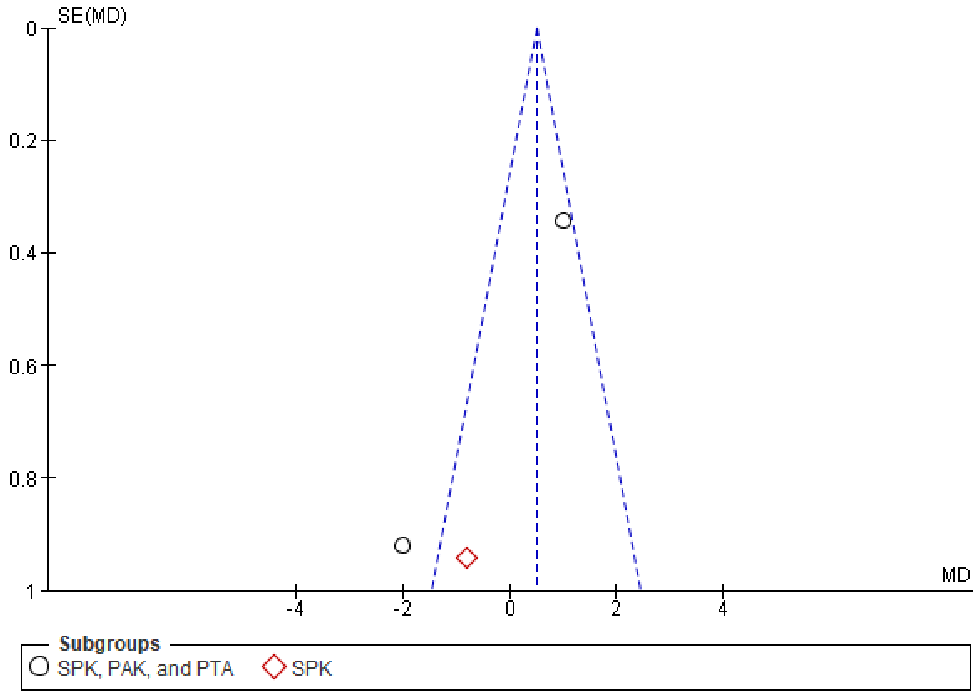

Supplement: Supplementary file 1 [file DataSheet1.pdf]
